# Salinomycin inhibits proliferative vitreoretinopathy formation in a mouse model

**DOI:** 10.1371/journal.pone.0243626

**Published:** 2020-12-21

**Authors:** Alison M. Heffer, Victor Wang, Richard T. Libby, Steven E. Feldon, Collynn F. Woeller, Ajay E. Kuriyan

**Affiliations:** 1 Flaum Eye Institute, University of Rochester, Rochester, NY, United States of America; 2 Center for Visual Sciences, University of Rochester, Rochester, NY, United States of America; 3 Retina Service, Wills Eye Hospital, Thomas Jefferson University, Philadelphia, PA, United States of America; Bascom Palmer Eye Institute, UNITED STATES

## Abstract

Proliferative vitreoretinopathy (PVR) is a progressive disease that develops in a subset of patients who undergo surgery for retinal detachment repair, and results in significant vision loss. PVR is characterized by the migration of retinal pigment epithelial (RPE) cells into the vitreous cavity, where they undergo epithelial-to-mesenchymal transition and form contractile membranes within the vitreous and along the retina, resulting in recurrent retinal detachments. Currently, surgical intervention is the only treatment for PVR and there are no pharmacological agents that effectively inhibit or prevent PVR formation. Here, we show that a single intravitreal injection of the polyether ionophore salinomycin (SNC) effectively inhibits the formation of PVR in a mouse model with no evidence of retinal toxicity. After 4 weeks, fundus photography and optical coherence tomography (OCT) demonstrated development of mean PVR grade of 3.5 (SD: 1.3) in mouse eyes injected with RPE cells/DMSO (vehicle), compared to mean PVR grade of 1.6 (SD: 1.3) in eyes injected with RPE cells/SNC (p = 0.001). Additionally, immunohistochemistry analysis showed RPE cells/SNC treatment reduced both fibrotic (αSMA, FN1, Vim) and inflammatory (GFAP, CD3, CD20) markers compared to control RPE cells/DMSO treatment. Finally, qPCR analysis confirmed that *Tgfβ*, *Tnfα*, *Mcp1* (inflammatory/cytokine markers), and *Fn1*, *Col1a1* and *Acta2* (fibrotic markers) were significantly attenuated in the RPE cells/SNC group compared to RPE/DMSO control. These results suggest that SNC is a potential pharmacologic agent for the prevention of PVR in humans and warrants further investigation.

## Introduction

Proliferative vitreoretinopathy (PVR) occurs in up to 10% of rhegmatogenous retinal detachments (RRDs) and is the leading cause of surgical failure, manifesting in poor visual outcomes and recurrent surgical interventions [1–4]. When a retinal tear occurs, inflammatory cytokines and growth factors are released into the vitreous cavity, which promote the dissociation and migration of retinal pigment epithelial (RPE) cells to the vitreous [5–7]. Some of the major cytokines associated with PVR and identified in vitreous samples of PVR patients include transforming growth factor beta (TGF-β), tumor necrosis factor alpha (TNF-α), platelet-derived growth factor alpha (PDGF-α), and monocyte chemotactic protein-1 (MCP1) [6–10]. Once in the vitreous, exposure to these cytokines and growth factors promotes epithelial-mesenchymal transition (EMT) of RPE cells to fibrotic cells, which may form membranes on either surface of the retina and within the vitreous. These membranes exerts traction on the retina, causing detachments and damage to the retinal photoreceptors [7,11,12].

Currently, there are no approved pharmacologic options for the inhibition of PVR formation. Surgical management involves removal of fibrotic membranes and in some cases excision of portions of the retina [4]. Pharmacologic agents that treat or prevent PVR formation would potentially improve surgical success rates and visual outcomes after retinal detachment. Molecules or compounds that target different processes involved in PVR pathology, including EMT, cell migration and contraction, would be potential promising therapies to treat or prevent PVR formation. The polyether ionophore salinomycin (SNC) was identified as a potent inhibitor of TGF-β driven myofibroblast (scar-cell) formation using a high-throughput screen [13]. SNC also blocked human capsular fibroblast-to-myofibroblast formation [14]. We recently showed that the SNC effectively inhibited several of the processes involved in PVR pathology *in vitro* in human RPE cells, including TGF-β-driven RPE cell migration, contraction of a collagen matrix and the process of EMT with no evidence of toxicity [15]. Others have shown that SNC inhibits TGFβ-driven EMT in cancer cells [16–19]. Based on these studies, we hypothesized that SNC would attenuate PVR formation *in vivo*. Here, we report that SNC is effective at reducing PVR formation in a mouse model of PVR [20]. Our results show that intravitreal SNC treatment inhibits development of PVR after 4 weeks compared to controls *in vivo*, demonstrating that SNC is a promising potential therapeutic agent for PVR that warrants further study.

## Results

### Salinomycin does not alter the structure or function of retinal cells

**Fig 1:**
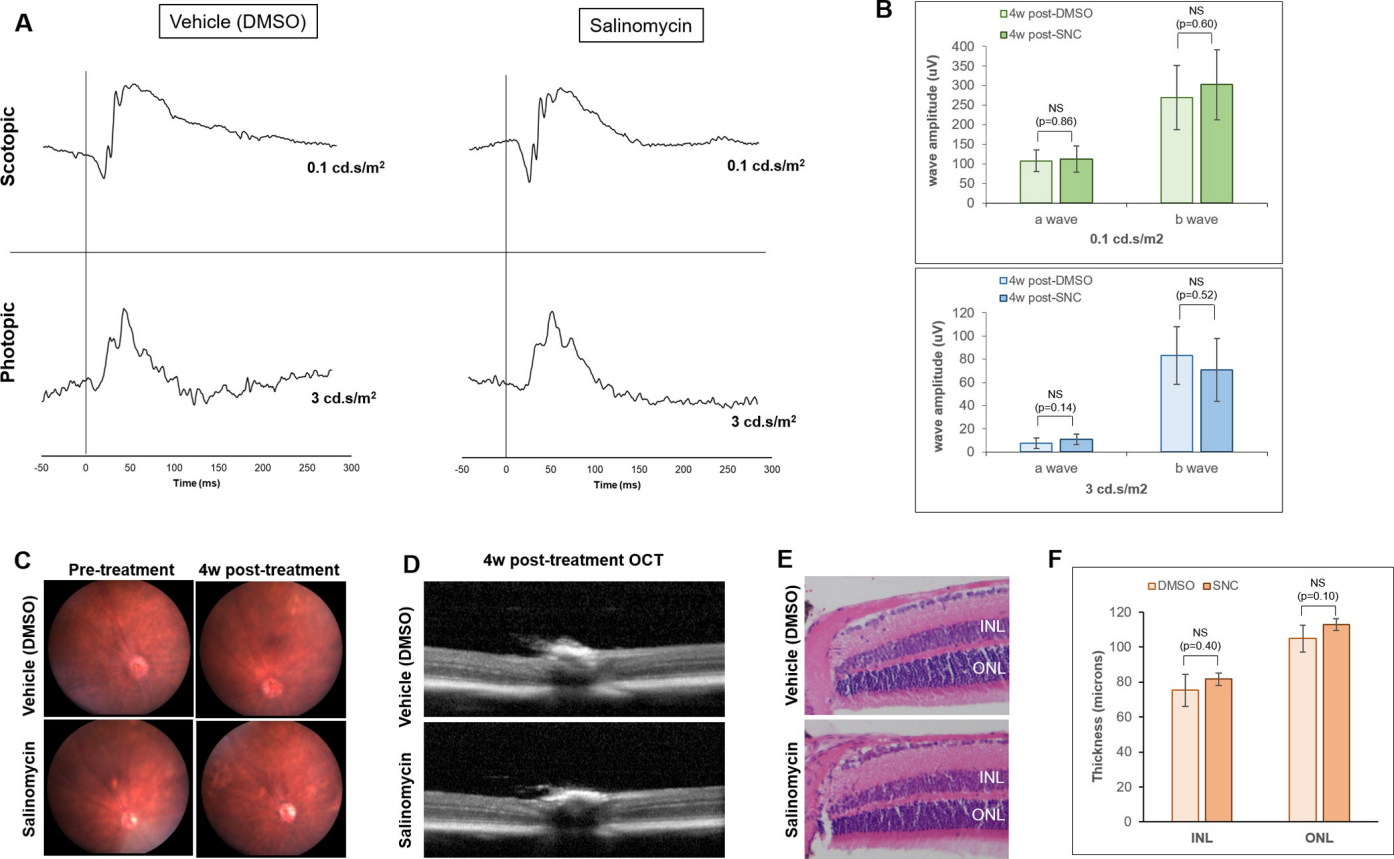
Salinomycin (SNC) does not significantly alter the function or structure of the rods and cones of the retina 4 weeks after intravitreal injection. A) DMSO-treated eyes have similar ERG traces to those treated with SNC for both scotopic (rod function) and photopic (cone function) parameters. B) The average a- and b-wave amplitudes are not significantly different between DMSO (n = 6) and SNC-treated (n = 9) eyes at scotopic and photopic light intensities. Fundus (C) and OCT (D) imaging reveals no differences in retinal appearance. E) H & E staining of the retina 4 weeks after treatment shows no differences in retina layers or appearance. F) The nuclear layer thicknesses for both the inner nuclear layer (INL) and outer nuclear layer (ONL) are not significantly different between DMSO-treated and SNC-treated eyes (n = 5 eyes for each analysis).

There was no significant difference between average a- and b-wave amplitudes 4 weeks after intravitreal vehicle control (DMSO) and 10μM salinomycin injections (Fig 1A and 1B). Additionally, imaging of the retina by both fundus photography and optical coherence tomography (OCT) show no abnormalities in retinal appearance (Fig 1C and 1D). Histology 4 weeks post-injection confirms that there are no abnormalities in the appearance of the retinal layers, cells or thickness of the outer and inner nuclear cell layers (Fig 1E and 1F). Together, these data support that salinomycin does not cause retinal toxicity when administered via intravitreal injection.

### A single intravitreal injection of salinomycin slows PVR formation in the mouse eye

**Fig 2:**
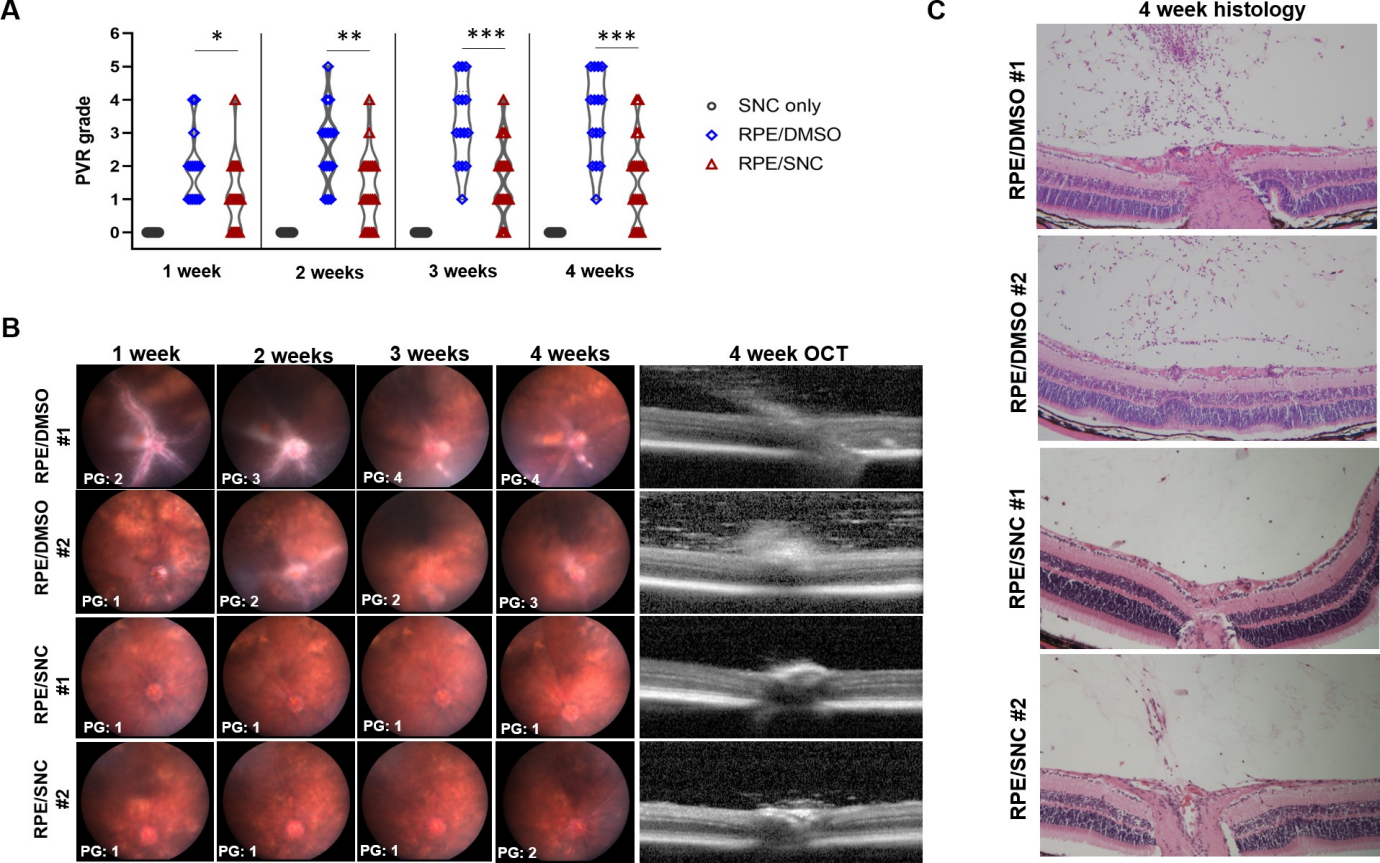
Salinomycin (SNC) is effective at slowing PVR progression over 4 weeks in a mouse model of PVR. A) A violin plot showing the distribution of PVR grades over 4 weeks for eyes injected with only SNC, RPE/DMSO and RPE/SNC. Mann-Whitney U tests showed significant differences between the distribution of PVR grades at all time-points (*p<0.05, **p<0.01, ***p<0.001). B) Representative weekly fundus images and a 4w OCT image from two representative eyes injected with RPE/DMSO and two representative eyes injected with RPE/SNC. C) H&E staining at 4 weeks post-treatment of the eyes injected with RPE/DMSO and RPE/SNC shown in B.

PVR development was compared between mice that received intravitreal 10μM SNC or DMSO (vehicle control) in conjunction with RPE cells by weekly fundus photography, along with OCT imaging and histological analysis after 4 weeks (Fig 2 and Table 1). After 1 week, mice that received intravitreal RPE/DMSO developed a mean PVR grade of 1.9 (SD: 1.0), which was significantly higher than mice that received intravitreal RPE/SNC (1.1, SD: 1.0, p = 0.026; Fig 2 and Table 2). 2 weeks after injection, the average PVR grade for eyes injected with RPE/DMSO (2.6, SD: 1.2) was also significantly higher than the average PVR grade for RPE/SNC injected eyes (1.3, SD: 1.1, p = 0.005; Table 2). The average PVR grades remained significantly higher at 3 weeks in RPE/DMSO eyes (3.3, SD: 1.3) compared to RPE/SNC eyes (1.6, SD: 1.1, p = 0.001; Table 2). Similarly at 4 weeks there was a higher average PVR grade in RPE/DMSO eyes (3.5, SD: 1.3) compared to RPE/SNC eyes (1.6, SD: 1.3, p = 0.001; Table 2). Importantly, none of the eyes injected with only SNC developed any PVR (Fig 2A). Together, these results show that a single intravitreal injection of 10μM SNC at the time of RPE cell injection potently inhibits the formation of PVR in the mouse eye over the course of 4 weeks. Of note, lower concentrations of intravitreal SNC (1μM and 5μM) were not effective at inhibiting PVR development compared to control.

**Table 1:** A summary of the key characteristics that define each grade of the mouse PVR model used in this study [20].

Mouse PVR Grade (PG)	Defining characteristics
0	• Clear vitreous • Normal retinal structures and vasculature
1	• Cells in vitreous • No points of retinal detachment
2	• Cells in vitreous and along retinal surface • Thickened retina, no detachments
3	• Cells forming tractional membranes • Small retinal folds and localized detachments
4	• Visible tractional membranes • Small retinal detachments (<25% detached)
5	• Tractional membranes and many retinal folds • Larger regions of retinal detachment (25–50%)
6	• Retina completely detached • Numerous retinal folds

**Table 2:** Intravitreal DMSO injection in the mouse PVR model resulted in significantly higher proportions of mice developing severe PVR compared to mice treated with intravitreal SNC injection at 2w, 3w, and 4w post-treatment. Any eyes in which optical opacities prevented imaging were excluded from analysis. Mann-Whitney U test showed significant differences in average PVR grades at all time-points; all values below p = 0.05 were considered significant.

Mean PVR Grade (SD)			p-value
	RPE/DMSO	RPE/SNC	
**1w**	1.9 (1.0)	1.1 (1.0)	0.026
**2w**	2.6 (1.2)	1.3 (1.1)	0.005
**3w**	3.3 (1.3)	1.6 (1.1)	0.001
**4w**	3.5 (1.3)	1.6 (1.3)	0.001

### Salinomycin inhibits the formation of PVR membranes in the vitreous and along the inner retinal surface

**Fig 3:**
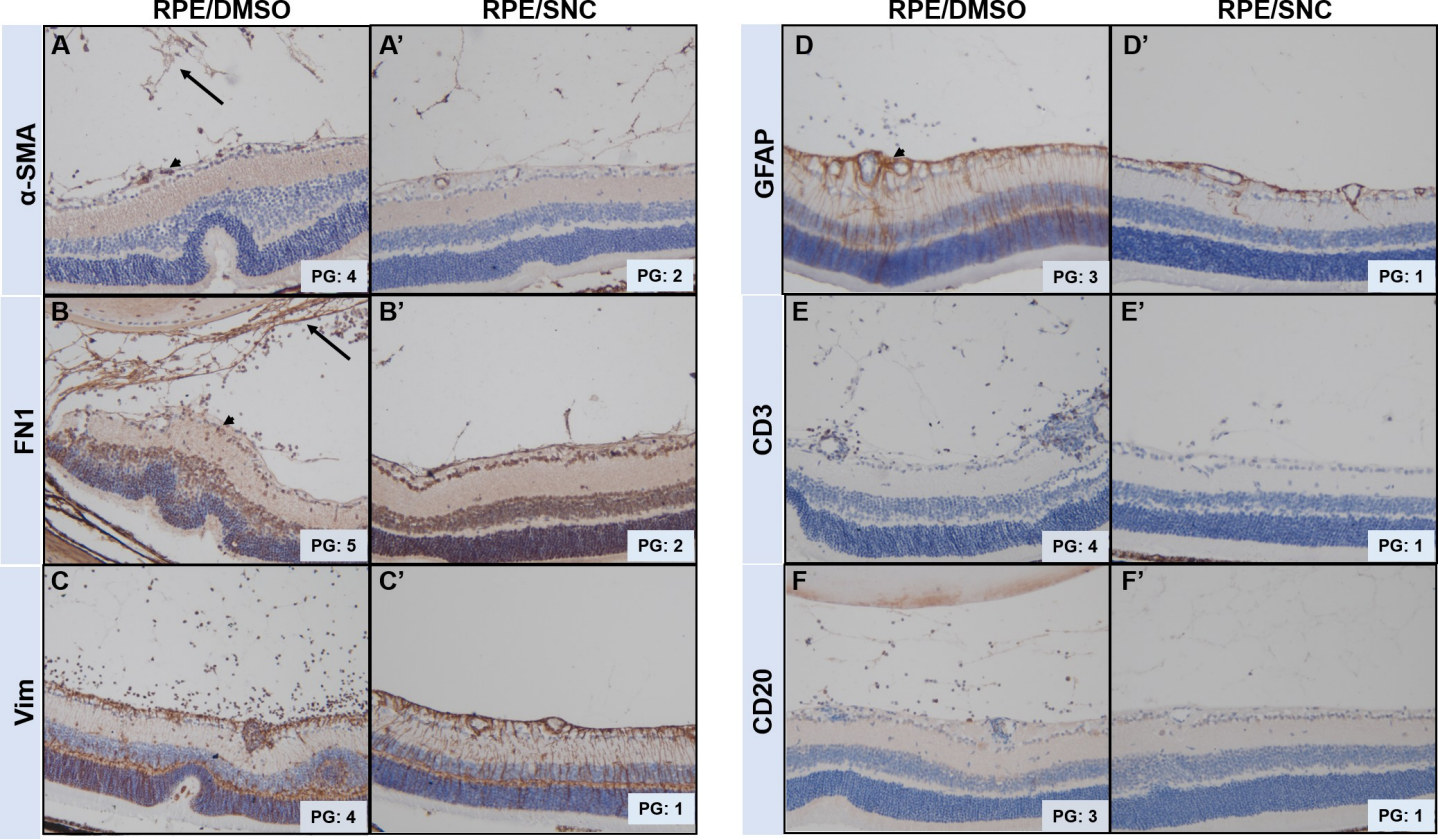
Mouse eyes treated with SNC show reduced formation of PVR membranes in the vitreous and along the inner retinal surface. Immunohistochemistry of markers (stained in brown) of fibrotic cells (A,B), glial cells (C,D), B-cells (E) and T-cells (F) are all expressed in the vitreous and along the retina surface of eyes treated with RPE/DMSO, but show reduced expression in eyes treated with RPE/SNC. Black arrow denotes PVR membranes in the vitreous and black arrowheads are PVR membranes along the retinal surface. PVR grades (PG) assigned to the eyes stained are shown.

To further support that intravitreal salinomycin treatment inhibited PVR formation at 4 weeks, we compared immunohistochemistry analysis of several known PVR markers within the vitreous and along the inner retinal surface in PVR mice eyes injected with DMSO and SNC (Fig 3). We found more cells and larger membranes in the vitreous and along the surface of the inner retina that more robustly expressed fibrotic markers, αSMA and FN1, in mice eyes that underwent intravitreal DMSO injection, compared to mice eyes that underwent SNC injection (Fig 3A and 3B). Similar differences were seen for Vimentin and GFAP proteins, which are expressed in activated glial cells, fibroblasts, and myofibroblasts (Fig 3C and 3D), and for CD3 and CD20, which are present on T and B cells, respectively (Fig 3E and 3F). Together, these immunohistochemical results show that SNC treatment results in inhibition of cell types that express markers of fibrotic, glial, B cells and T cells, which are all components of human PVR membranes [12,21,22].

### Salinomycin treatment reduces gene expression of markers associated with PVR

**Fig 4:**
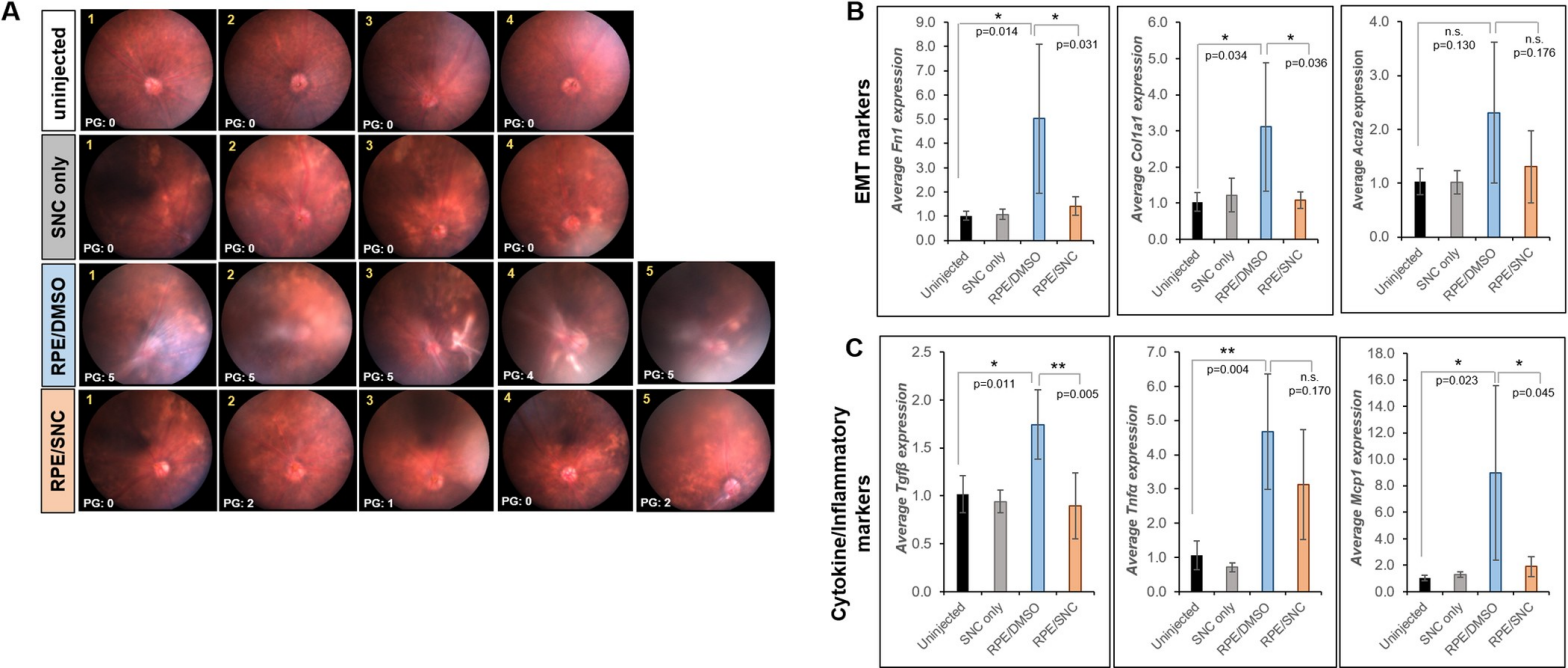
qPCR analysis of mouse eyes treated with SNC show reduced expression of PVR markers. A) 4 week fundus images with PVR grades (PG) of each eye used for analysis. Average gene expression of B) EMT markers (*Fn1*, *Col1a1* and *Acta2*) and C) cytokines and inflammatory markers (*Tgfβ*, *Tnfα*, and *Mcp1*) all show an overall up-regulation in mouse eyes injected with RPE/DMSO (blue bars) after 4 weeks compared to eyes injected with RPE/SNC (orange bars). Uninjected (black bars) and eyes injected with only SNC (grey bars) did not show up-regulation of these genes. Each bar represents the average fold change from all eyes in that treatment groups. Significance between uninjected and RPE/DMSO and RPE/DMSO and RPE/SNC was determined using a one-way ANOVA test followed by Tukey’s post-hoc analysis. *: p<0.05; **: p<0.01; n.s.: not significant.

We compared the effects of RPE/DMSO and RPE/SNC injection by quantitative PCR (qPCR) of fibrotic markers (*Fn1*, *Col1a1* and *Acta2*) and chemokines/cytokines (*Mcp1*, *Tnfα*, and *Tgfβ*) that have been associated with PVR development in the whole eye 4 weeks post-treatment. Since biological variation was expected, we conducted this analysis in 4 uninjected eyes, 4 eyes injected with only SNC, and 10 eyes with varying degrees of PVR induced by RPE injection (5 treated with DMSO and 5 treated with SNC, Fig 4A). The fibrotic markers *Fn1* and *Col1a1* had statistically significantly ~5-fold (p = 0.014) and ~3-fold (p = 0.034) increase, respectively, in eyes with RPE/DMSO injection compared to eyes that received no injection (Figs 4B and S1). There was a statistically significant reduction to baseline levels for both genes with RPE/SNC injection (p = 0.031 for *Fn1*, p = 0.036 for *Col1a1*; Fig 4B). While overall average *Acta2* (αSMA gene) levels were higher in the eyes treated with RPE/DMSO compared to uninjected eyes, the difference was not significant (~2.3-fold; p = 0.130; Figs 4B and S1). Average *Acta2* transcript levels were lower with RPE/SNC treatment, though not statistically significant from RPE/DMSO levels (p = 0.176; Figs 4B and S1). There was a 1.7-fold increase of *Tgfβ* transcripts in RPE/DMSO injected eyes compared to controls (p = 0.011); this was statistically significantly decreased with RPE/SNC treatment (p = 0.005; Figs 4C and S1). *Tnfα*, a cytokine involved in inflammation, showed a 4.7-fold upregulation in eyes that received intravitreal injection of RPE/DMSO compared to uninjected eyes (p = 0.004; Figs 4C and S1). While intravitreal injection of RPE/SNC resulted in a lower overall average of *Tnfα* transcripts, this difference was not significant from RPE/DMSO treatment (p = 0.170; Fig 4C). Additionally, another marker associated with inflammation, *Mcp1*, was also significantly upregulated in eyes injected with RPE/DMSO compared to controls (p = 0.023; Figs 4C and S1). Injection of RPE/SNC showed a significant reduction in *Mcp1* levels compared to RPE/DMSO, close to controls (p = 0.045; Fig 4C). Importantly, there was little biological variation of transcript levels in uninjected eyes, and none of the eyes injected with salinomycin only showed upregulation of these PVR markers (S1 Fig). Together, these results confirm a significant upregulation of many genes associated with PVR in humans upon RPE/DMSO treatment in a mouse model and importantly, this upregulation is inhibited by RPE/SNC treatment.

## Discussion

Identification of effective pharmacologic agents that inhibit the formation of PVR is a critical unmet need and can potentially improve visual and surgical outcomes after retinal detachments. To date, there are no prospective clinical trials that have identified agents that consistently inhibit PVR formation [23–28].

Salinomycin was first isolated from *Streptomyces albus*, and has been used as an antibiotic in agricultural feed and also in nutrient absorption for several decades [29,30]. In recent years, SNC has been explored as a treatment for different types of cancers [19,31,32] and scar formation [13,33]. It has also been reported to reduce chronic inflammation [34].

We previously identified SNC as a potential agent to inhibit PVR based on its ability to inhibit multiple aspects of PVR pathogenesis *in vitro*, including RPE cell EMT, contraction, and migration [15]. Others have also reported its effectiveness in inhibiting TGFβ-induced EMT [17,19,35], a known mechanism driving PVR formation. TGFβ is one of the major molecular drivers of RPE cell EMT in PVR and has been found in the vitreous of PVR patients [8,36–38]. It was previously shown in cell culture that SNC could inhibit TGFβ-induced EMT of both RPE cells [15] and myofibroblasts [13]. Our new *in vivo* studies presented here suggest that SNC may reduce PVR formation by inhibition of EMT processes. While we did not study the mechanism(s) whereby SNC blocks PVR *in vivo*, our previous *in vitro* studies found that SNC inhibits RPE cell TGFβ-induced EMT through early inhibition of non-canonical TGFβ signaling, via inhibition of phospho-p38 expression and later inhibition of canonical TGFβ signaling, via phospho-Smad2 [15]. A similar mechanism whereby SNC attenuated phosphorylation of p38, SMAD2 and TGFβ activated kinase 1 (TAK1) was identified in human orbital fibroblasts [13]. Therefore, we hypothesize these pathways play a role in the ability of SNC to block PVR *in vivo*. In addition to these targeting these pathways, SNC targets the β-catenin/Wnt signaling cascade [39–41]. Specifically, SNC blocked β-catenin transcriptional activity by preventing β-catenin/TCF4 complex formation in colorectal cancer cells. In tumor xenograph studies, SNC also reduced expression of Wnt target genes including CD44, Sox2 and LGR5. Wnt signaling also appears to be key in EMT observed in PVR [9]. Interestingly, fibronectin is also a Wnt target gene and we show that SNC potently attenuates fibronectin accumulation and gene expression in PVR *in vivo*. Thus, future studies investigating whether SNC can block the β-catenin/Wnt signaling cascade in PVR are warranted.

In this study, we report that a single intravitreal injection of the polyether ionophore salinomycin in a mouse model of PVR was sufficient to inhibit formation of PVR. Importantly, SNC did not appear to have any toxicity on the structure or function of retina cells after intravitreal injection, as it did not cause changes in either the appearance of the retina or the function of rods and cones in the photoreceptors, determined through ERG (Fig 1).

The current study demonstrates that *in vivo* SNC intravitreal injection inhibits several EMT markers (fibronectin, collagen 1, vimentin, GFAP, and αSMA) known to be expressed in human PVR membranes (Figs 3 and 4) [42,43]. FN1 is a major component of the extracellular matrix of PVR membranes found within the vitreous and along retinal surfaces of PVR patients, whose contraction results in concurrent retinal detachments [44]. Additionally, FN1 was found to be one of the most abundant transcripts in an analysis of human PVR epiretinal membranes [45]. Vimentin and Col1A1 transcripts were upregulated in human PVR membranes [45], and Vimentin levels were also found to be increased in a rabbit model of PVR [46]. Glial fibrillary acidic protein (GFAP) has also been detected in epiretinal membranes removed from PVR patients [42].

Additionally, in this study we analyzed expression of αSMA, an early marker of EMT. We saw a clear increase in αSMA expression along the inner retinal surface and within the vitreous by immunostaining, which was visibly less abundant with SNC treatment (Fig 3). However, qPCR analysis did not show significant upregulation of *Acta2* transcripts with RPE/DMSO injection or a significant difference between transcript levels in RPE/DMSO and RPE/SNC injection (Fig 4). The inability to detect significant changes in *Acta2* levels in RPE/DMSO-injected eyes was likely due to the fact that our analysis included transcripts of the entire eye, and *Acta2* is also expressed in the lens at high levels [43], likely masking the change we see in the histological analysis (Fig 3). Clearly, PVR induction in the mouse eye up-regulates expression of many known EMT markers, all of which are reduced when SNC is administered at the time of PVR induction.

In addition to EMT markers, we also found multiple inflammatory cells and cytokines known to be involved in PVR pathogenesis are inhibited by SNC treatment (Figs 3 and 4), including the T cell marker CD3 and B cell marker CD20 [47], as well as one of the main regulators of monocytes and macrophages, MCP1 [10]. Another cytokine involved in inflammation and upregulated PVR eyes is *Tnfα* [48] (Fig 4). While we saw a significant upregulation of *Tnfα* transcripts in RPE/DMSO-injected mouse eyes, SNC treatment did not yield a significant reduction of *Tnfα* transcripts. However, the levels of *Tnfα* in the RPE/SNC eyes were also not statistically significantly higher than the uninjected eyes (p = 0.115). There was a large amount of biologic variability in the amount of *Tnfα* transcripts RNA and it is possible that with a much larger sample size we would have been able to detect statistically significant differences.

SNC has shown great promise in targeting various types of cancer cells and blocking scar-formation. While no clinical trials are ongoing in the US, several small human case studies have been performed in Central and South America [30]. These reports have shown that SNC intravenous infusion has been effective in treating disease with minimal side effects. Interestingly, in PVR, SNC could be given intravitreally, which could minimize systemic exposure. The mounting evidence of SNC’s effectiveness in multiple disease processes makes it a promising candidate drug for future early stage clinical studies.

One limitation of the current paper is that SNC was administered at the same time as RPE cells in the PVR model. This application tests the ability of SNC to inhibit PVR formation, not treat PVR that has already formed. Such an approach is commonly used when testing candidate agents for PVR in animal models [49]. This approach was also employed in our study to mimic one of the few human studies with positive results for PVR: the use of oral isotretinoin for prevention of PVR development, not treatment of formed PVR membranes [50]. In that study, there was no difference in surgical success rate among eyes that already had PVR at the time of surgery between patients who received oral isotretinoin and historical controls (78.4% versus 70.0%, p = 0.358). However, there was a significantly better surgical success rate among patients who had clinical features that were high risk for the development of PVR and received oral isotretinoin, compared to historical controls (84.5% versus 61.1%, p = 0.005). The results from our study support the potential of studying intravitreal SNC in a similar manner, in patients with high risk clinical features. Of note, the systemic side effects of isotretinoin, including its teratogenic effects, have resulted in limited adoption of isotretinoin by retina specialists [50].

In summary, our study demonstrates that intravitreal SNC is a potential pharmacologic agent to inhibit development of PVR without evidence of retinal toxicity. The treatment modality of intravitreal injection limits the degree of systemic side effects. Our findings support the importance of early clinical trials for SNC to further evaluate its ability to prevent PVR formation in humans with high risk RRDs.

## Materials and methods

### Animals

Six to eight week old female C57BL/6J mice were purchased from Jackson Laboratory (Bar Harbor, ME). All experiments adhered to the ARVO Statement for the Use of Animals in Ophthalmic and Vision Research and were approved by the University Committee of Animal Resources (UCAR) of the University of Rochester.

### Electroretinography (ERG) measurements

Mice were anesthetized by intraperitoneal injection of 100mg/kg ketamine (Par Pharmaceuticals, Chestnut Ridge, NY) and 10 mg/kg xylazine (Akorn Inc, Lake Forest, IL). 1μL 10μM SNC (n = 4 mice) or DMSO (n = 2 mice) was administered into the eye by intravitreal injection. After 4 weeks, retinal function was assessed using the Celeris ERG system specific for rodents (Diagnosys, Lowell MA). Prior to imagining, mice were dark-adapted for 18 hours. Dark-adapted ERG measurements were recorded at 0.1cd.s/m^2^ to analyze rod function, followed by exposure at a light intensity of 3 cd.s/m^2^ to measure cone function. Average a- and b-wave amplitudes for all mice injected with DMSO or SNC were determined and no significant differences were found between treatment groups using a Mann-Whitney U statistical analysis. Thickness of the inner and outer nuclear layers was calculated by taking the average of 3 measurements 100 microns apart at a location ~400 microns from the optic nerve. The measurement tool on the Gryphax imaging software (Jenoptiks, Jena, Germany) was used to calculate nuclear thicknesses and the Mann-Whitney U statistical test was used to determine significance between treatment groups.

### PVR induction and salinomycin treatment

PVR was induced in one eye of the mouse as previously reported [20]. Briefly, mice were anesthetized by intraperitoneal injection of 100mg/kg ketamine (Par Pharmaceuticals, Chestnut Ridge, NY) and 10 mg/kg xylazine (Akorn Inc, Lake Forest, IL). A posterior vitreous detachment was induced by intravitreal injection of 0.5μL SF_6_ gas (Alcon Laboratories, Ft. Worth, TX). One week later, immediately prior to injection, freshly harvested RPE cells (ARPE-19, ATCC, Manassas VA) at a concentration of 4x10^4^ cells per microliter were mixed with 20μM salinomycin (S4526; Sigma, St. Louis MO) or DMSO (vehicle control) for 2 minutes, resulting in a 10μM SNC solution that contained 2x10^4^ RPE cells per microliter. 1μL of this solution was injected intravitreally. The RPE cell/treatment mixture was injected slowly and the needle was left in the eye for 30 seconds after RPE injection to prevent cells from leaking upon needle removal. In total, 15 eyes were injected with RPE/DMSO and 20 eyes were injected with RPE/SNC. These injections were done in groups of 5 eyes per treatment (experimental replication) and at different times (biological replication). All PVR grades were determined using the Mouse PVR Grading Scale [20]. Any eyes that had cataracts or other media opacities which prevented retinal imaging were not included in the analysis.

### Ocular imaging

#### Fundus photography

Eyes were examined by fundus photography weekly to monitor the development of PVR. Mice were anesthetized as described above and pupils were dilated using an ophthalmic solution of phenylephrine 2.5% (Paragon Bioteck Inc, Portland, OR) and tropicamide 1% (Akorn Inc, Lake Forest, IL). GenTeal lubrication gel (Alcon, Fort Worth, TX) was applied to prevent ocular surface drying. Eyes were imaged using the bright-field view of the Micron III (Phoenix Instruments, Naperville, IL) and images were acquired using StreamPix software (Norpix, Montreal, Quebec).

#### OCT

Optical coherence tomography (OCT) imaging was performed 4 weeks after injection of RPE cells +/- SNC to assess retinal structures and membranes resulting from PVR development. Mice were anesthetized and the pupil was dilated as described above. A small contact lens was placed on the eye to improve the optics and prevent the ocular surface from drying. OCT images were captured using the Heidelberg Spectralis HRA+OCT imaging system (Heidelberg Engineering, Franklin, MA).

### Histology and immunohistochemistry

#### Hematoxylin and eosin (H+E) staining

Mouse eyes were fixed in 4% paraformaldehyde for 24 hours at 4°C, dehydration through a series of ethanol washes and then embedded in paraffin. 10-micron sections were cut using a Microm HM310. Hematoxylin and eosin (H+E) staining was performed as described previously [20].

#### Immunohistochemistry

Eyes were fixed, embedded in paraffin and sectioned as described above. After deparaffinization, antigen retrieval was performed by in citrate buffer (pH 6) for 5 minutes in the microwave. Slides were blocked in 10% goat serum/1% BSA for 1–2 hours at room temperature, and then incubated overnight in primary antibody diluted in 1% BSA/TBS. After a 15 minute incubation in 0.3% H_2_O_2_, an HRP-conjugated secondary antibody diluted in 1% BSA was applied for 2 hours at room temperature. Slides were stained with DAB (Vector Laboratories) and counterstained with hematoxylin before mounting. All imaging was done on an Olympus BX51 microscope (Olympus, Shinjuku, Tokyo, Japan). Primary antibodies and dilutions used were αSMA (1:250, rabbit, Abcam, Cambridge, UK), FN1 (1:500, rabbit, Abcam), Vim (1:500, rabbit, Cell Signaling Technologies, Danvers, MA), GFAP (1:500, rabbit, Cell Signaling Technologies), CD3 (1:250, rabbit, GeneTex, Irvine, CA), CD20 (1:250, rabbit, LSBio, Seattle, WA). For the secondary antibody, an HRP-conjugated secondary antibody (1:500, goat, Jackson ImmunoResearch, West Grove, PA) was used.

### Quantitative PCR (qPCR) analysis of PVR transcripts

Whole eyes were dissected and placed into TRIzol (Invitrogen, Carlsbad CA). After homogenizing, total RNA was extracted per the manufacturer’s protocol. RNA quantification and quality were determined using a NanoDrop 1000. cDNA was made with the QuantiTect Reverse Transcription Kit (Qiagen, Hilden Germany) per manufacturer’s instructions, and 25ng was used as a template in each reaction. All primer sets were designed using IDT’s Real Time qPCR Tool and amplified a region spanning an exon-exon boundary and are listed below. *Gapdh* was used as a control.

**Table:** 

Gene	Forward primer	Reverse primer
*Gapdh*	5' ATG CCA TCA CTG CCA CCC AG	5' GGG ATG ACC TTG CCC ACA GC
*Fn1*	5' GGA GGA AAT AGC CCT GTC CA	5' CGG CCA GTG ACA GCA TAC A
*Col1a1*	5' CCT GGA CAG CCT GGA CTT CC	5' AGG GAG ACC ACG AGG ACC AGA
*Acta2*	5' GCA GGT CAT CAC CAT CGG C	5' TGA TGC TGT TGT AGG TGG TCT C
*Mcp1*	5' GTC CCT GTC ATG CTT CTG G	5' GCT CTC CAG CCT ACT CAT TG
*Tnfα*	5' CTT CTG TCT ACT GAA CTT CGG G	5' CAG GCT TGT CAC TCG AAT TTT G
*Tgfβ*	5' CCT GAG TGG CTG TCT TTT GA	5' CGT GGA GTT TGT TAT CTT TGC TG

Quantitative PCR (qPCR) was performed using SsoAdvanced Universal SYBR Green Supermix and protocol (Bio-Rad, Hercules CA). Primer sets were run in triplicate with each cDNA. Expression levels of all genes for each eye were normalized to GAPDH levels in that eye. To calculate fold change, the comparative CT method was used [51]. For each gene, the ΔCT calculated in the 4 un-injected eyes was averaged and used to calculate the fold change for each eye that received SNC only, RPE/DMSO and RPE/SNC. Error bars for each eye were obtained from the technical replicates for each gene. The qPCR data has been deposited on Figshare (DOI 16.6084/m9.figshare.13079537).

### Statistical analysis

Fundus and OCT images of mice were graded at 1, 2, 3 and 4 weeks post-RPE injection using a PVR grading scheme for mice. While no masked analysis was used for determining the PVR of each animal, there was agreement for all assigned grades by three individuals familiar with the model, one of whom is a retinal specialist. The distribution of grades for each injection group are presented as violin plots and Mann-Whitney U tests were used to compare the severity of PVR grade between groups at each time point. All p-values < 0.05 were considered significant. For determining statistical significance in the qPCR experiments, one-way ANOVA tests followed by Tukey’s post-hoc tests were done. All p-values < 0.05 were considered significant.

## Supporting information

S1 Fig Quantitative PCR (qPCR) of transcripts involved in PVR. 4 uninjected eyes (black bars), 4 eyes injected with SNC only (grey bars), 5 eyes injected with RPE/DMSO (blue bars), and 5 eyes injected with RPE/SNC (orange bars) were used for transcript level analysis for early fibrotic genes (*Fn1*, *Col1a1*, and *Acta2*), a cytokine (*Tgfβ*), and inflammatory markers (*Tnfα* and *Mcp1*). Each bar represents the average fold change from three technical replicates for one animal, normalized to *Gapdh* and compared to uninjected eyes (see Materials and Methods).
(TIF)
