# Supplementary figures and images for: Salinomycin inhibits proliferative vitreoretinopathy formation in a mouse model
Source: PLoS One. 2020 Dec 21;15(12):e0243626. doi: 10.1371/journal.pone.0243626 (PMC7751870; doi:10.1371/journal.pone.0243626)

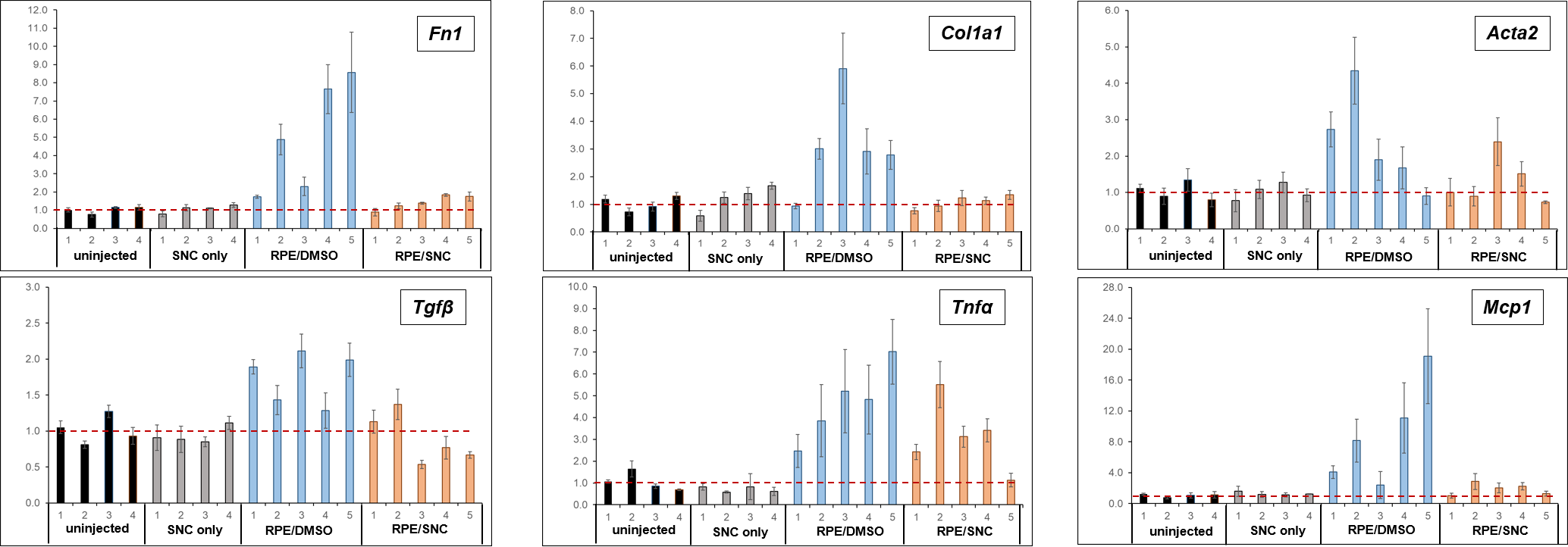

Supplement: S1 Fig — 4 uninjected eyes (black bars), 4 eyes injected with SNC only (grey bars), 5 eyes injected with RPE/DMSO (blue bars), and 5 eyes injected with RPE/SNC (orange bars) were used for transcript level analysis for early fibrotic genes (Fn1, Col1a1, and Acta2), a cytokine (Tgfβ), and inflammatory markers (Tnfα and Mcp1). Each bar represents the average fold change from three technical replicates for one animal, normalized to Gapdh and compared to uninjected eyes (see Materials and Methods). (TIF) [file pone.0243626.s001.tif]
